# Risk Stratification for the Rate and Location of Residual Bladder Tumor for the Decision of Re-Transurethral Resection of Bladder Tumor

**DOI:** 10.3389/fonc.2022.788568

**Published:** 2022-01-27

**Authors:** Junjie Fan, Xing Zhang, Jinhai Fan, Lei Li, Dalin He, Kaijie Wu

**Affiliations:** ^1^ Department of Urology, First Affiliated Hospital of Xi’an Jiaotong University, Xi’an, China; ^2^ Department of Urology, Baoji Central Hospital, Baoji, China

**Keywords:** re-transurethral resection of bladder tumor, bladder cancer, residual tumor, urothelial carcinoma subspecialist, detrusor muscle

## Abstract

**Introduction:**

To assess the rate and location of residual tumor in re-transurethral resection of bladder tumor (re-TURBT) and develop a risk stratification tool to assist clinicians in making treatment decisions.

**Patients and Methods:**

The data of 144 patients with high-risk bladder cancer who received re-TURBT were retrospectively reviewed. The rate and location of residual tumors was recorded. Logistic regression was performed to explore risk factors for residual tumors, and a risk classification tool was developed.

**Results:**

Among the 144 patients, the rates of residual tumor and tumor location at the base of the primary tumor were 22.2% and 10.4%, respectively. Non-urothelial carcinoma subspecialist, piecemeal resection and the absence of detrusor muscle in the first specimen were defined as risk factors. Patients were categorized into low-, intermediate-, and high-risk groups according to the number of risk factors. The rate of residual tumor in the high-risk group was significantly higher than that in the low- and intermediate-risk groups (50% *vs.* 7.8%, *P*=0.001; 50% *vs.* 18.6%, *P*=0.002). Moreover, high-risk patients benefitted more from a second resection at the base of the primary tumor due to the high rate of residual tumor located at this site than low- and intermediate-risk patients (23.5% *vs.* 2.0%, *P*=0.002; 23.5% *vs.* 10.2%, *P*=0.083).

**Conclusions:**

Risk stratification based on the subspecialist category, operative method, and presence or absence of detrusor muscle in the first specimen could help identify patients who benefit from re-TURBT and second resection the base of the primary tumor.

## Introduction

Bladder cancer (BCa) is the ninth most common cancer worldwide and ranks 13th in terms of annual mortality from cancer (1, 2). Transurethral resection of bladder tumor (TURBT) followed by intravesical adjuvant chemotherapy or immunotherapy is the standard diagnostic and treatment method for non-muscle invasive bladder cancer (NMIBC) (3, 4). However, TURBT represents a challenge for urologists due to the high incidence of residual tumors (5, 6). A systemic review that contained 31 studies on 8409 patients with NMIBC revealed that the incidence of residual tumor was 17-67% in patients with Ta and up to 20-71% in patients with T1 after first TURBT (7).

Residual tumor following TURBT has been considered to be partly responsible for recurrence (8), and the European Association of Urology (EAU) guidelines recommend re-TURBT for patients with high-risk BCa (9). During re-TURBT, the base of the primary tumor should be second resected by the operating surgeon to eradicate residual disease and ensure accurate pathological staging. However, the incidence of residual tumor in re-TURBT specimens is low, especially when detrusor muscle (DM) is present in the first TURBT specimen. In addition, re-TURBT may impose an additional economic and emotional burden on patients, and a second resection at the base of the primary tumor will increase the risk of bladder perforation, especially for women (10).

Furthermore, a systematic meta-analysis of six studies detailing 3257 participants recently showed that re-TURBT did not improve survival outcomes in patients with T1 BCa (11). Similarly, a retrospective study conducted by Gontero et al. noted that in patients with high-grade T1 BCa treated with intravesical BCG, re-TURBT did not improve oncological outcomes (12). Furthermore, Calò et al. also documented that re-TURBT did not bring a survival benefit in patients with completely resected high-risk BCa (13).

These findings raise questions regarding the necessity of re-TURBT and second resection of the base of the primary tumor in patients with high-risk BCa. Thus, identifying patients who might benefit from re-TURBT and second resection of the base of the primary tumor would be very valuable. In the present study, we aimed to assess the rate and location of residual tumors in re-TURBT specimens and to explore the risk factors. Moreover, a risk stratification tool was developed to identify patients who would likely benefit from re-TURBT and second resection at the base of the primary tumor.

## Materials and Methods

### Study Population

We retrospectively reviewed the medical records of patients who received re-TURBT at our institute between 2013 and 2019. The inclusion criteria were as follows: (1) met the indications for re-TURBT according to the EAU or the American Urological Association guidelines (9, 14); (2) the re-TURBT included the resection of all visible tumors and areas with a scar, oedema and the base of the primary tumor; (3) re-TURBT were performed by urothelial carcinoma (UC) subspecialists. Patients with a history of upper tract urothelial carcinoma or prostatic stroma invasion in the first TURBT specimen as well as patients with incomplete data were excluded. After reviewing the medical data in our institute, 186 patients received re-TURBT from 2013 to 2019. However, 42 patients were excluded, including 19 patients with incomplete data, 9 patients with a history of upper tract urothelial carcinoma and 14 patients whose re-TURBT were performed by the non-urothelial carcinoma (UC) subspecialists. Thus, 144 patients were finally included.

### Clinicopathological Evaluation

The presence and location of residual tumors were confirmed by experienced pathologists through a histologic review of the re-TURBT specimen. Tumor stage, grade, diameter and numbers in the first TURBT were confirmed by pathologists and urologists. Tumor stage was determined according to the 2009 TNM classification, and pathological grade was determined according to the 2004 World Health Organization (WHO) classification. Urologists were classified into UC subspecialists and non-UC subspecialists according to the annual surgery volume of urothelial carcinoma. UC subspecialists had completed fellowship training in urothelial cancer and performed more than 300 operations of urothelial carcinoma each year, including TURBT, radical cystectomy, partial cystectomy and radical nephroureterectomy, which was significantly more than non-UC subspecialists.

### Statistical Analysis

Data on continuous variables are presented as the mean ± standard deviation, and differences between different groups were analyzed with Student’s t-test. The optimal cut-off points for tumor diameter and the number of tumors and time period between first TURBT and re-TURBT were calculated by receiver operating characteristic (ROC) curves based on the largest Youden index. Differences in the categorical variables between different groups were determined using Pearson’s chi-squared test or Fisher’s exact test as appropriate. Logistic regression models were used to assess the independent risk factors for residual tumor. Statistical analysis was performed using PASW Statistics 18.0 (formerly SPSS, Chicago, IL, USA) and GraphPad Prism software (GraphPad Software, La Jolla, CA, USA). P<0.05 was considered to indicate a significant difference.

## Results

### Baseline Characteristics of the Patients

The baseline characteristics of the 144 included patients are shown in **Table 1**. The mean age was 62.26 ± 10.59 years, and 108 (75.0%) patients were men. The majority of first TURBT procedures (71.5%) were performed by UC subspecialists. Moreover, 81.2% of patients chose bipolar TURBT as the operative method, while 18.8% chose front-firing potassium-titanyl-phosphate (KTP) green-light laser *en bloc* resection.

**Table 1:** Clinicopathologic characteristics of the patients who underwent re-TURBT.

Characteristic	
Total patients	144
Age, years (mean ± SD)	62.26 ± 10.59
Sex [patients (%)]	
Male	108 (75.0%)
Female	36 (25.0%)
Recurrence status [patients (%)]	
Primary	127 (88.2%)
Recurrence	17 (11.8%)
Operative method of first TURBT [patients (%)]	
KTP laser	27 (18.8%)
Bipolar TURBT	117 (81.2%)
Operator of first TURBT [patients (%)]	
UC subspecialist	103 (71.5%)
Non-UC subspecialist	41 (28.5%)
Tumor diameter [patients (%)]	
< 3 cm	99 (68.8%)
≥ 3 cm	45 (31.2%)
Tumor number [patients (%)]	
< 3	67 (46.5%)
≥ 3	77 (53.5%)
T stage of the first TURBT specimen [patients (%)]	
Ta	9 (6.2%)
T1	135 (93.8%)
Pathologic grade of the first TURBT specimen [patients (%)]	
Low-grade	18 (12.5%)
High-grade with or without variant histology	126 (87.5%)
DM present in the first TURBT specimen [patients (%)]	
No	99 (68.8%)
Yes	45 (31.2%)
Time between first TURBT and re-TURBT	
≤6 weeks	92 (63.9%)
>6 weeks	52 (36.1%)
Residual tumor presence in the re-TURBT specimen [patients (%)]	
No	112 (77.8%)
Yes	32 (22.2%)
Residual tumor site [patients (%)]	
Base of the primary tumor	11 (34.4%)
Resection margins and excision scar	16 (50.0%)
New lesion	1 (3.1%)
Base of the primary tumor, resection margins and excision scar	2 (6.3%)
Base of the primary tumor and new lesion	1 (3.1%)
Base of the primary tumor, resection margins, excision scar and new lesion	1(3.1%)

DM, detrusor muscle; KTP, front-firing potassium-titanyl-phosphate; UC, urothelial carcinoma; TURBT, transurethral resection of bladder tumor.

The pathologic stage and grade distributions at first TURBT were as follows: there were 9 patients with pTa (6.2%) and 135 with pT1 (93.8%), and there were 18 patients with low-grade (12.5%) and 126 with high-grade with or without variant histology (87.5%). DM was present in the first TURBT specimen in 45 patients. Furthermore, 67 patients (46.5%) had fewer than 3 lesions, and 99 patients (68.8%) presented with small lesions (diameter<3 cm). Ninety-two (63.9%) patients received re-TURBT within 6 weeks after the first TURBT, and residual tumor was found in 32 patients (22.2%). Moreover, 9 patients (6.25%) with lymphovascular invasion (LVI), 18 patients (12.50%) with histological variants, and only 28 patients (19.44%) with carcinoma *in situ* (CIS) were identified based on the first TURBT specimens. All patients received postoperative continued bladder washing (**Supplementary Table 1**).

### Locations and Risk Factors for Residual Tumors in Re-TURBT

The distribution of residual tumor in re-TURBT specimens is shown in **Table 1**. Among the 32 patients who had residual tumors in re-TURBT specimens, 11 (34.4%) had tumors at the base of the primary tumor, 16 (50.0%) had tumors at the resection margin and a scar from excision of the primary tumor, and 1 (3.1%) had a new lesion. Furthermore, 2 patients (6.3%) had tumors at the base of the primary tumor, resection margins and a scar from excision of the primary tumor, and 1 (3.1%) had tumors at the base of the primary tumor and new lesions. In addition, residual tumor at the base of the primary tumor, resection margins, a scar from excision of the primary tumor and a new lesion was found in 1 patient (3.1%). In total, 15 patients (46.9%) had a residual tumor at the base of the primary tumor, and in 93.3% of these patients, DM was not present in the first TURBT specimen.

A comparison of patients with and without residual tumor after their first TURBT is shown in **Table 2**. The residual tumor rate did not display any significant difference when assessing most of the clinicopathological characteristics. Moreover, there was no differences of the residual tumor in terms of LVI, histological variants, CIS and primary tumor site (**Supplementary Table 1**). However, the residual tumor rate was significantly lower in patients treated by UC subspecialists and in those with DM in the first TURBT specimen (P<0.05). The logistic regression analysis revealed that surgery performed by non-UC subspecialists and the absence of DM in the first TURBT specimen was associated with residual tumor in the re-TURBT specimen (**Table 3**). First TURBT performed by non-UC subspecialists was associated with the presence of residual tumor in the re-TURBT specimen (odds ratio [OR]: 8.782; 95% confidence interval [CI]: 3.66-21.071, *P*=0.001). The absence of DM in the first TURBT specimen was also associated with the presence of residual tumor in the re-TURBT specimen. Specifically, the risk was increased by 3-fold when DM was absent in the first TURBT specimen.

**Table 2:** Comparison of patients with and without residual tumor in re-TURBT specimens.

	With residual tumor in the re-TURBT specimen (n = 32)	Without residual tumor in the re-TURBT specimen (n = 112)	*p*
Age, years (mean ± SD)	62.25 ± 11.75	62.26 ± 10.30	0.921
Sex [patients (%)]			0.643
Male	25 (23.1%)	83 (76.9%)	
Female	7 (19.4%)	29 (80.6%)	
Recurrence status [patients (%)]			0.448
Primary	27 (21.3%)	100 (78.7%)	
Recurrence	5 (29.4%)	12 (70.6%)	
Operative method of first TURBT [patients (%)]			0.304
KTP laser	4 (14.8%)	23 (85.2%)	
Bipolar TURBT	28 (23.9%)	89 (76.1%)	
Operator of first TURBT [patients (%)]			0.001
UC subspecialist	11 (10.7%)	92 (89.3%)	
Non-UC subspecialist	21 (51.2%)	20 (48.8%)	
Tumor diameter [patients (%)]			0.387
< 3 cm	24 (24.2%)	75 (75.8%)	
≥ 3 cm	8 (17.8%)	37 (82.2%)	
Tumor number [patients (%)]			0.448
< 3	13 (19.4%)	54 (80.6%)	
≥ 3	19 (24.7%)	58 (75.3%)	
T stage of the first TURBT specimen [patients (%)]			0.999
Ta	2 (22.2%)	7 (77.8%)	
T1	30 (22.2%)	105 (77.8%)	
Pathologic grade of the first TURBT specimen [patients (%)]			0.225
Low-grade	6 (33.3%)	12 (66.7%)	
High-grade with or without variant histology	26 (20.6%)	100 (79.4%)	
DM present in the first TURBT specimen [patients (%)]			0.031
No	27 (27.3%)	72 (72.7%)	
Yes	5 (11.1%)	40 (88.9%)	
Time between first TURBT and re-TURBT			0.817
≤6 weeks	21 (22.8%)	71 (77.2%)	
>6 weeks	11 (21.2%)	41 (78.8%)	

DM, detrusor muscle; KTP, front-firing potassium-titanyl-phosphate; UC, urothelial carcinoma; TURBT, transurethral resection of bladder tumor.

**Table 3:** Logistic regression analyses of the association between residual tumor in re-TURBT specimens and clinicopathologic characteristics.

	OR	95% CI	*P*
Age (Continuous)	0.999	0.963-1.038	0.997
Sex (Male *vs*. Female)	1.248	0.488-3.190	0.644
Recurrence status (Primary *vs*. Recurrence)	1.543	0.500-4.761	0.45
Operator of first TURBT (UC subspecialist *vs*. Non-UC subspecialist)	8.782	3.66-21.071	0.001
Operative method of first TURBT (Bipolar TURBT *vs*. KTP laser)	1.809	0.576-5.676	0.31
Tumor diameter (< 3 cm *vs*. ≥ 3 cm)	0.676	0.277-1.648	0.389
Tumor number (< 3 *vs*. ≥ 3)	1.361	0.613-3.019	0.449
T stage of the first TURBT specimen (Ta *vs*. T1)	0.999	0.197-5.068	0.999
Pathologic grade of the first TURBT specimen (Low-grade *vs*. High-grade with or without variant histology)	0.52	0.178-1.517	0.231
DM present in the first TURBT specimen (No *vs*. Yes)	3	1.072-8.399	0.036
Time between first TURBT and re-TURBT (≤6 weeks *vs*. >6 weeks)	0.907	0.398-2.069	0.817

DM, detrusor muscle; KTP, front-firing potassium-titanyl-phosphate; UC, urothelial carcinoma; TURBT, transurethral resection of bladder tumor.

### Construction of the Risk Stratification Tool

Based on the above findings, we constructed a risk stratification model to assist urologists in identifying well-selected patients who will benefit from re-TURBT. Because KTP green-light laser *en bloc* resection was associated with the presence of DM in the first TURBT specimen (OR: 2.467, 95% CI: 1.046-5.814; *P*=0.039) (**Supplementary Table 2**), piecemeal resection of the tumor from the first TURBT was also considered a risk factor in the stratification, in addition to the absence of DM in the first TURBT specimen and non-UC subspecialists.

**Figure 1:**
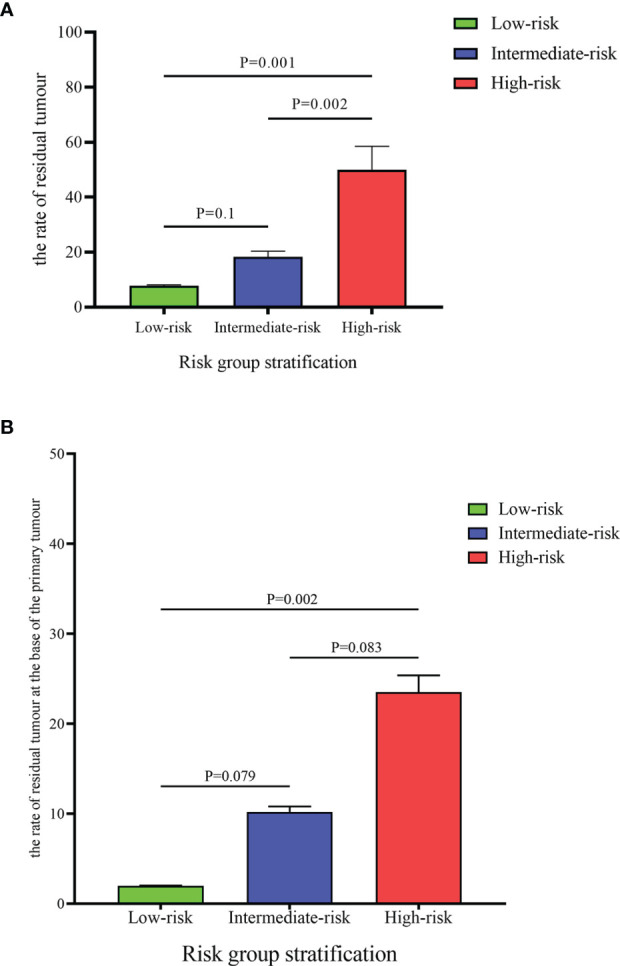
Comparison of the rate of residual tumour **(A)** and the rate of residual at the base of the primary tumour **(B)** between different risk groups.

According to the presence of risk factors, patients were assigned to three groups. Patients with no or one risk factor were assigned to the low-risk group (51 patients: 35.42%), those with two risk factors were assigned to the intermediate-risk group (59 patients: 40.97%), and all the other patients (with three risk factors) were assigned to the high-risk group (34 patients: 23.61%). As shown in **Table 4**, the rate of residual tumor at any location or base of the primary tumor was significantly different between different risk groups (*P*<0.05). Moreover, the rate of residual tumor in the high-risk group was significantly higher than that in the low- and intermediate-risk groups (50% *vs.* 7.8%, *P*=0.001; 50% *vs.* 18.6%, *P*=0.002) (**Figure 1A**). Furthermore, residual tumor was more likely located at the base of the primary tumor in the high-risk group than in the low-risk group (23.5% *vs.* 2.0%, *P*=0.002). The difference in the rate of residual tumor at the base of the primary tumor between the intermediate-risk group and the low- or high-risk group was almost statistically significant (10.2% *vs.* 2.0%, *P*=0.079; 10.2% *vs.* 23.5%, *P*=0.083) (**Figure 1B**).

**Table 4:** Rate of residual tumor at any location or base of the primary tumor in different risk groups.

	Low-risk group (n = 51)	Immediate-risk group (n = 59)	High-risk group (n = 34)	*P*
Residual tumor				0.001
No	47 (92.2%)	48 (81.4%)	17 (50.0%)	
Yes	4 (7.8%)	11 (18.6%)	17 (50.0%)	
Residual tumor at the base of the primary tumor				0.006
No	50 (98.0%)	53 (89.8%)	26 (76.5%)	
Yes	1 (2.0%)	6 (10.2%)	8 (23.5%)	

## Discussion

Re-TURBT is an important part of the optimal management of high-risk BCa and is recommended by several international guidelines. However, several recent publications suggest that re-TURBT could be avoided in well-selected patients with high-risk BCa and identifying them would be very valuable (12, 15, 16).

The presence of DM in resection samples is a suitable indicator of complete resection and indirectly reflects the quality of the first TURBT. Dutta et al. showed that staging inaccuracy was critically dependent on the absence of DM in the specimen, with upstaging at radical cystectomy in 62% and 30% of patients without or with DM during TURBT, respectively (17). Furthermore, a retrospective study conducted by Huang et al. revealed that the rate of residual tumor was 51.8% in patients without DM in the first TURBT specimen, which was significantly higher than that in patients with DM in the first specimen (51.8% *vs.* 20.9%, OR: 15.537, 95% CI: 2.814-85.789, *P*=0.002) *(* 18). Similarly, Ayati et al. documented that the risk of residual tumor was increased by 21-fold when DM was absent in the first TURBT specimen (19). Moreover, they found that the absence of DM in the first resection specimen was associated with upstaging (OR: 8.123, 95% CI: 1.478-44.632), indicative of the presence of residual tumor at the base of the primary tumor. Subsequently, our study also confirmed that the absence of DM in the first resection specimen was associated with the risk of residual tumor, and this residual tumor was likely to be located at the base of the primary tumor (OR: 7.247, 95% CI: 0.923-56.926, *P*=0.06) (**Supplementary Table 3**). In addition, a recent study revealed that re-TURBT may not be necessary in patients with T1-HG/G3 if DM is present in the first TURBT specimen (12). These results strongly indicate that the absence of DM in the first resection is an important surrogate marker of residual tumor. Patients without DM in the first TURBT specimen should receive re-TURBT, and surgeons should ensure that DM is obtained from the base of the primary tumor to confirm the depth of invasion during re-TURBT.

The adequacy and completeness of TURBT depend on the experience of the surgeon (20). In a retrospective study, Zurkirchen et al. analyzed the data of 214 patients treated with re-TURBT and found urologists in training had an equally low rate of residual tumor compared to senior urologists (27% *vs.* 37%, *P*=0.08) *(* 21). However, various studies have documented that senior surgeons are likely to achieve a higher rate of DM presence and decrease the residual tumor rate (22–24). To our knowledge, this was the first study to explore the relationship between residual tumor and the expertise of surgeons. Furthermore, we found that UC subspecialists are more likely to achieve clean resection than non-UC subspecialists. The major reason for this phenomenon is that the UC subspecialists were experienced and confident in performing resections that were sufficiently wide and deep while ensuring technical safety. Another reason is that UC subspecialists were more likely to obtain DM in the first resection (OR: 8.721, 95% CI: 2.525-30.118, *P*=0.001) (**Supplementary Table 2**). Moreover, the risk of residual tumor located at the base of the primary tumor increased by 4.5-fold when the first resection was performed by non-UC subspecialists (**Supplementary Table 3**). These results indicate that the surgeon’s experience and expertise are important indicators of the rate and location of residual tumors.

*En bloc* resection possesses a better hemostatic effect, clearer surgical fields of vision, and high-quality histological specimens than TURBT, as the integrity and architecture of the tumor can be maintained. Kramer et al. showed that DM was found in specimens of 97.3% of patients who underwent *en bloc* resection for a bladder tumor (25). Moreover, various studies have demonstrated that *en bloc* resection can result in a high rate of DM presence (theoretically up to 100%) and complete tumor removal (24, 26, 27). The same conclusion was reached in the present study: *en bloc* resection was associated with the presence of DM (OR: 2.467, 95% CI: 1.046-5.814; *P*=0.039) (**Supplementary Table 2**). All these results suggest that *en bloc* resection can achieve high-quality resection and may decrease the number of re-TURBT procedures.

Recently, the management of NMIBC has been improved by new endoscopic technologies, such as photodynamic diagnosis (PDD) and narrow-band imaging (NBI). Owing to the advantage of tumor visualization, PDD and NBI could improve the quality of TURBT and may be promising technologies to avoid unnecessary re-TURBT. A literature review that included 44 studies showed that the rate of residual disease after PDD resection was only 4.5–32.7% compared to 25.2–53.1% after white-light resection (28). Furthermore, they also pointed out that the odds ratio of residual tumor for PDD was 0.28 compared to white-light but the relative risk of residual disease was 2.77-fold compared to white-light. Moreover, Ma et al. analyzed the data of 124 patients with NMIBC, and found that NBI-assisted TURBT could significantly reduce the rate of residual disease (29). However, a recent study showed that the rate of residual tumor was as high as 58.7% at the second TURBT with PDD (30). Thus, whether PDD or NBI could avoid re-TURBT still remains unconcluded and further studies are required.

Multiparametric magnetic resonance imaging (mp-MRI) for BCa could provide a high tissue contrast resolution and effectively differentiate bladder wall layers (31, 32). Thus, mp-MRI can assist urologists to identify the infiltration depth of tumor. Giudice et al. prospectively collected the data of 231 patients who underwent mp-MRI before initial TURBT and found that the sensitivity, specificity of mp-MRI to identify patients with MIBC at re-TURBT was 85% (95% CI: 62-96.8%) and 93.6% (95% CI: 86.6-97.6), respectively (33). Moreover, a study also documented that mp-MRI with a vesical imaging reporting and data system (VI-RADS) was an effective and reliable method to determine the patients who could benefit from re-TURBT (34). Furthermore, in the era of big data and precision medicine, not only mp-MRI, but also artificial intelligence (35) and molecular biomarkers (36) have become promising tools to identify patients with BCa who will benefit more from re-TURBT.

In our study, the rate of residual tumor during re-TURBT was 22.2% (32/144), and only 15 patients (10.4%) had a residual tumor at the base of the primary tumor. Several previous studies have reported that residual tumors are found in up to 50% of patients (17, 37), and between 30% and 60% of T1G3 BCa will become muscle invasive at radical cystectomy (38), which was higher than was identified in our study. The complete preoperative evaluation in our institution, including the acquisition of tumor characteristics and lesion biopsy during the preoperative cystoscopy examination, as well as the selection of an appropriate operative method, is the major reason. Moreover, these results indicate that a proportion of high-risk BCa patients may not benefit from re-TURBT and second resection at the base of the primary tumor. The patients in our study were categorized into risk groups based on the expertise of the surgeon, the operative method, and the presence or absence of DM in the first specimen. Half of the patients in the high-risk group had a residual tumor at re-TURBT, which was significantly higher than that of patients in the low- and intermediate-risk groups. Moreover, the rate of residual tumor at the base of the primary tumor was still higher in the high-risk group than in the low- and intermediate-risk groups (23.5% *vs.* 2.0% *vs.* 10.2%). Hence, these patients could benefit from re-TURBT and second resection at the base of the primary tumor.

Similar to other retrospective studies, this study was limited by a retrospective study design and a small sample size from a single center, which might lead to a selection bias. Furthermore, the role of this risk stratification as a diagnostic tool for candidates for re-TURBT was not externally validated. Therefore, further prospective multicenter studies are warranted to support our findings.

## Conclusion

Non-UC subspecialists and the absence of DM in the first TURBT specimen are risk factors for residual tumor at re-TURBT. Furthermore, en bloc resection may improve the rate of DM presence in the first TURBT specimen. Risk stratification based on the above three factors may help identify patients who might benefit from re-TURBT and second resection the base of the primary tumor.

## Data Availability Statement

The datasets presented in this study can be found in online repositories. The names of the repository/repositories and accession number(s) can be found in the article/**Supplementary Material**.

## Ethics Statement

Written informed consent was obtained from the individual(s) for the publication of any potentially identifiable images or data included in this article.

## Author Contributions

JJF: data collection, data analysis, and manuscript writing. XZ: data collection and manuscript writing. JHF: performed operations and manuscript editing. LL: performed operations and manuscript editing. DH: project development and manuscript editing. KW: project development, performed operations, and manuscript editing. All authors contributed to the article and approved the submitted version.

## Funding

This study was supported by the Clinical Research Award of the First Affiliated Hospital of Xi’an Jiaotong University, China (No. XJTU1AF-CRF-2015-002 to DH).

## Conflict of Interest

The authors declare that the research was conducted in the absence of any commercial or financial relationships that could be construed as a potential conflict of interest.

## Publisher’s Note

All claims expressed in this article are solely those of the authors and do not necessarily represent those of their affiliated organizations, or those of the publisher, the editors and the reviewers. Any product that may be evaluated in this article, or claim that may be made by its manufacturer, is not guaranteed or endorsed by the publisher.
