# Supplementary material for: Risk Stratification for the Rate and Location of Residual Bladder Tumor for the Decision of Re-Transurethral Resection of Bladder Tumor
Source: Front Oncol. 2022 Jan 27;12:788568. doi: 10.3389/fonc.2022.788568 (PMC8829133; doi:10.3389/fonc.2022.788568)
Supplement: Supplementary file 1 [file DataSheet_1.docx]

Supplementary Material

# Supplementary Tables

**Supplement Table 1** Histological information of first TURBT and the relationship between the rate of residual tumour in the re-TURBT specimen

|  | With residual tumour in the re-TURBT specimen (N=32) | Without residual tumour in the re-TURBT specimen (N=112) | Total(N=144) | *P* |
| --- | --- | --- | --- | --- |
| LVI present in the first TURBT specimen [patients (%)] |  |  |  | 0.408 |
| Yes | 3 (33.33%) | 6 (66.67%) | 9 (6.25%) |  |
| No | 29 (21.48%) | 106 (78.52%) | 135 (93.75%) |  |
| Isthological variant present in the first TURBT specimen [patients (%)] |  |  |  | 0.544 |
| Yes | 5 (27.78%) | 13 (72.22%) | 18 (12.50%) |  |
| No | 27 (21.43%) | 99 (78.57%) | 126 (87.50%) |  |
| Concomitant CIS in the first TURBT specimen [patients (%)] |  |  |  | 0.694 |
| Yes | 7 (25.00%) | 21 (75.00%) | 28 (19.44%) |  |
| No | 25 (21.55%) | 91 (78.45%) | 116 (80.56%) |  |
| Post operative continue bladder washing [patients (%)] |  |  |  | 0.999 |
| Yes | 32 (22.22%) | 112 (77.78%) | 144 (100%) |  |
| No | 0 | 0 | 0 (0%) |  |
| The site of primary BCa [patients (%)] |  |  |  | 0.923 |
| Trigone | 12 (20.69%) | 46(79.31%) | 58 (9.97%) |  |
| Right wall | 16 (22.86%) | 54 (77.14%) | 70 (12.03%) |  |
| Left wall | 21 (22.34%) | 73 (77.66%) | 94 (16.15%) |  |
| Anterior wall | 25 (25.25%) | 74 (74.75%) | 99 (17.01%) |  |
| Posterior wall | 26 (22.81%) | 88 (77.19%) | 114 (19.59%) |  |
| Dome | 28 (28.28%) | 71 (71.72%) | 99 (17.01%) |  |
| Neck | 10 (20.83%) | 38 (79.17%) | 48 (8.25%) |  |

LVI: lymphovascular invasion; CIS: carcinoma in situ; TURBT: transurethral resection of bladder tumour; BCa: bladder cancer;

**Supplement Table 2** Logistic regression analyses for the association between DM presence in first TURBT specimen and clinicopathologic characteristics

|  | OR | 95%CI | *P* |
| --- | --- | --- | --- |
| Age (Continuous) | 0.992 | 0.960-1.026 | 0.651 |
| Sex (Female vs. Male) | 1.044 | 0.461-2.364 | 0.917 |
| Recurrence status (Primary vs. Recurrence) | 1.549 | 0.476-5.046 | 0.467 |
| Operator of first TURBT (Non-UC subspecialist vs. UC subspecialist) | 8.721 | 2.525-30.118 | 0.001 |
| Operative method of first TURBT (Bipolar TURBT vs. KTP laser) | 2.467 | 1.046-5.814 | 0.039 |
| Tumour diameter (< 3 cm vs. ≥ 3 cm) | 0.751 | 0.355-1.587 | 0.453 |
| Tumour number (< 3 vs. ≥ 3) | 1.13 | 0.557-2.294 | 0.735 |
| T stage of the first TURBT specimen (Ta vs. T1) | 0.611 | 0.122-3.066 | 0.55 |
| Pathologic grade of the first TURBT specimen (Low-grade vs. High-grade with or without variant histology) | 0.592 | 0.183-1.912 | 0.381 |
| Time between first TURBT and re-TURBT (≤6 weeks vs. >6 weeks) | 0.684 | 0.332-1.412 | 0.304 |

DM: detrusor muscle; KTP: front-firing potassium-titanyl-phosphate; UC: urothelial carcinoma; TURBT: transurethral resection of bladder tumour

**Supplement Table 3** Logistic regression analyses for the association between residual tumour at the base of the primary tumour in re-TURBT specimen and clinicopathologic characteristics

|  | OR | 95%CI | *P* |
| --- | --- | --- | --- |
| Age (Continuous) | 0.978 | 0.929-1.031 | 0.408 |
| Sex (Female vs. Male) | 2.326 | 0.499-10.844 | 0.282 |
| Recurrence status (Primary vs. Recurrence) | 3.245 | 0.902-11.669 | 0.071 |
| Operator of first TURBT (UC subspecialist vs. Non-UC subspecialist) | 4.547 | 1.502-13.763 | 0.007 |
| Operative method of first TURBT (KTP laser vs. Bipolar TURBT) | 3.534 | 0.444-28.114 | 0.233 |
| Tumour diameter (< 3 cm vs. ≥ 3 cm) | 0.78 | 0.234-2.599 | 0.686 |
| Tumour number (< 3 vs. ≥ 3) | 6.602 | 1.432-30.432 | 0.015 |
| T stage of the first TURBT specimen (Ta vs. T1) | 0.926 | 0.108-7.956 | 0.944 |
| Pathologic grade of the first TURBT specimen (Low-grade vs. High-grade with or without variant histology) | 0.526 | 0.133-2.081 | 0.36 |
| DM present in the first TURBT specimen (Yes vs. No) | 7.247 | 0.923-56.926 | 0.06 |
| Time between first TURBT and re-TURBT (≤6 weeks vs. >6 weeks) | 1.146 | 0.370-3.555 | 0.813 |

DM: detrusor muscle; KTP: front-firing potassium-titanyl-phosphate; UC: urothelial carcinoma; TURBT: transurethral resection of bladder tumour
